# The Evolving Role of Nurses in Hospital Settings—A Scoping Review

**DOI:** 10.1111/jan.70345

**Published:** 2025-11-11

**Authors:** Mia Björk, Irene Eriksson, Anette Ekström‐Bergström, Viola Nyman

**Affiliations:** ^1^ University West Trollhättan Trollhättan Sweden; ^2^ NU‐Hospital Group Trollhättan Trollhättan Sweden

**Keywords:** accountability, adaptability, development, leadership, learning, motivation, responsibility

## Abstract

**Aim:**

To explore existing research regarding how nurses' unclear responsibilities influence their professional role development in hospital settings.

**Design:**

A scoping review was conducted according to Arksey and O'Malley.

**Methods:**

The Population Exposure Outcome framework was used to identify eligible inclusion and exclusion criteria and search terms. The included articles have been thematically analysed in guidance by Braun and Clarke.

**Data Sources:**

Twenty‐six studies conducted between 2016 and 2024 were identified in CINAHL, PubMed and Scopus.

**Results:**

The roles of nurses were highly adaptable within hospital settings and healthcare teams, requiring nurses to assess both organisational shortcomings and colleagues' needs. These assessments depended on the nurses' competence and motivation for professional growth.

**Conclusion:**

There was a clear need to strengthen organisational structures and frameworks to support the evolving role of nurses. Basic nursing education must be better at preparing nurses for their upcoming leadership role.

**Impact:**

Increasing complexity and unclear responsibilities contribute to role ambiguity among nurses. The review highlights the importance of recognising nurses as visible and accountable leaders. There is also a need to support nurses' adaptability through improved basic education, which may have implications for both clinical practice and education.

**Reporting Method:**

The PRISMA Scoping Review checklist was used during the review process. The PRISMA flowchart was used to report the database searches.

**Patient or Public Contribution:**

This study did not include patient or public involvement in its design, conduct, or reporting.

**Summary**

- Contribution to the wider global clinical community
  ○ Nurses should be recognised as visible leaders who have redefined responsibilities grounded in accountability.
  ○ Basic education must emphasise adaptability and leadership to prepare nurses for their evolving roles.
- What is already known
  ○ Nurses' roles have become increasingly complex and unclear due to nursing shortages and the redistribution of tasks within interdisciplinary teams worldwide.
- What this paper adds
  ○ Expanding and ambiguous responsibilities are reshaping nurses’ roles, which highlights the need for aligned education and organisational support to ensure safe care and clearly defined professional boundaries.
- Implications for practice
  ○ Healthcare organisations should clarify nurses’ roles, promote the safe delegation of tasks, and provide leadership training to support nurses’ professional growth, accountability, and teamwork.

## 1 Introduction

Nurses are at the forefront of patient care and represent the largest professional group within the global healthcare workforce (WHO 2025). Their responsibilities and ethical codes define, shape and set boundaries for their professional roles and legal obligations (Grace et al. 2024). However, worldwide nursing shortages and rapidly transforming healthcare contexts across diverse countries have changed the way that care is organised and responsibilities are distributed among healthcare professions (Grace et al. 2024; Grover and Fritz 2022; Kagonya et al. 2023). These shifts are not confined to a single healthcare system; instead, they constitute a shared international challenge, with implications for patient safety, workforce sustainability and the need to strengthen nursing to reach health‐related Sustainable Development Goals (WHO 2025). In this transforming context, nurses' responsibilities have become increasingly complex and unclear. For example, responsibilities may be rearranged in healthcare teams, where direct patient care could be performed by non‐licensed professionals, such as assistant nurses (Kagonya et al. 2023; Björk et al. 2024). Because of changing assignments, it is necessary to clarify expectations regarding others' responsibilities in the healthcare team in order to avoid tensions and uncertainty between the professionals (Björk et al. 2024). Studies have also shown that expanding or delegating responsibilities between professions requires scrutiny of the effects on the entire healthcare system, both at individual and organisational levels (Niezen and Mathijssen 2014). For instance, to reorganise responsibilities in healthcare teams, it is necessary to have supportive regulations and frameworks to clarify expectations and assure patient safety (National Health Care Competence Council [NHCCC] 2024).

Fawcett (2021) summarised how different ways of organising nursing care affect clarity of accountability in patient care. Accountability is seen as distinct from responsibility, as the former involves taking responsibility for one's actions and behaviours, but also one's inactions. Accountability involves lifelong learning and continuous professional growth, but also delivering high‐quality care, upholding professional standards and being prepared to justify actions and failures to those affected (Krautscheid 2014). Fawcett (2021) compared a functional, task‐focused nursing model with a team nursing model, where the latter could sacrifice individual accountability and allow negative outcomes (Fairbrother et al. 2015; Fawcett 2021). Nurses remain accountable for delegated nursing assignments, as supervision of assistant nurses' performance may prevent adverse events (Hughes et al. 2017; Wagner 2018). Accordingly, rearranging responsibilities in healthcare is not easy, as it requires management, support and structure to clarify responsibilities (Grover and Fritz 2022; Saga et al. 2024).

Such rearrangements also impact nurses' professional role (NHCCC 2024). Nurses' professional roles and responsibilities are defined as being guided by a nursing perspective while functioning in an interdisciplinary field (American Association of Colleges of Nursing [AACN] 2021). The role has shifted from assisting physicians to an autonomous profession with its own expertise in nursing (Hall 1997). This evolution relates to growing nursing research and demographic changes in society (WHO 2025). Modern nurses must be adaptable, skilled in specialised patient‐centred care and able to work in rapidly changing environments. Their role increasingly includes leadership and care coordination in collaborative teams (Alshammari et al. 2024), which requires strong communication skills and the ability to support other professionals (Grace and Uveges 2023). Globally, nurses' roles and titles vary widely, showing differences in responsibilities, competencies and expectations on performance (WHO 2025). In support of the World Health Organisation, Baker et al. (2020) argued that demographic changes require a high standard of nursing education to meet future needs, ensuring disciplinary knowledge aligns with expected competencies (AACN 2021). Hence, as healthcare continues to change, the nurses' role must evolve. These arguments stress lifelong learning and possible retraining as important for adjusting competences and skills to new prerequisites (Notarnicola et al. 2025).

In summary, the literature highlights the increasing unclarity and complexity of nurses' responsibilities, leading to unclear expectations of their role and a need for learning to provide safe care. Over the past century, nurses' roles have developed from physician support to an autonomous, multifaceted profession, where accountability informs the complexity of nursing responsibilities. Modern nurses are crucial contributors in patient care and are expected to provide individualised, specialised care, while taking on leadership and coordination roles in interdisciplinary teams. These increased expectations, combined with complex patient needs, underscore the importance of understanding the evolution of nurses' roles in contemporary healthcare settings. However, more research is needed to understand how unclear responsibilities influence nurses' role development. Given that health systems worldwide are reorganising professional roles in response to workforce shortages and demographic changes, this represents an international concern with significant implications for nursing education, regulation and clinical practice.

## 2 Aim

This review aimed to explore existing research regarding how nurses' unclear responsibilities influence their professional role development in hospital settings.

## 3 Design

### 3.1 Scoping Review

This scoping review used the methodology described by Arksey and O'Malley (2003). We felt that a scoping review was valuable for identifying the existing and available research in the specific research area but could also be used to identify key factors related to a concept, in this case nursing responsibilities and the nursing role (Munn et al. 2018). No previous protocol has been created concerning this scoping review. The review process has followed the PRISMA Scoping Review (ScR) checklist (Tricco et al. 2018), added in Attachment IV.

#### 3.1.1 Stage 1—Identifying the Research Question

A Population Exposure Outcome (PEO) framework (see Table 1) was seen as appropriate for the scoping review because it provided structure to the research question, which is based on the review aim (Bettany‐Saltikov and McSherry 2016).

**TABLE 1:** The PEO framework based on the research question.

How does unclear nursing responsibilities influence the role development of nurses in hospital settings?	
P: Population	Nurses in hospital settings
E: Exposure	Unclear nursing responsibilities
O: Outcome	Influence on the role development of nurses

### 3.2 Data Collection

#### 3.2.1 Stage 2—Identifying Relevant Studies

The search strategy was based on the PEO framework/research question, inclusion/exclusion criteria and limitations. The systemic process when doing a review is the most important way to ensure credibility and avoid bias when reporting the results, starting with the search strategy, drawing on guidance from Sandelowski (2008):

1. Consulting the university librarian
2. Decision of databases with the librarian
3. Creating search blocks/search terms and inclusion/exclusion criteria based on the PEO framework
4. Decision of combinations of search blocks
5. Pilot test
6. Re‐test and revise search terms
7. Adjustment after databases (MeSH, free‐text, thesaurus, subject headings, Boolean operators AND/OR and truncations)

After consulting the university librarian, four databases were chosen. The databases were chosen based on the research question to gain a broad coverage of studies: PubMed (health‐related research), CINAHL (nursing research), Scopus (broader interdisciplinary range), and EBSCO ERIC (pedagogical and educational‐related research). The inclusion and exclusion criteria were developed in consultation with the research team. Here, the PEO framework based on the study aim was used as guidance when deciding the inclusion and exclusion criteria (see Table 2). After this, search blocks were developed based on the PEO framework. A pilot test was conducted on January 17, 2025 in Scopus. After this pilot search, the inclusion and exclusion criteria and the following search terms were revised until the most optimal search terms for the upcoming study were selected; for example, both the E (Exposure) and O (Outcome) needed several clarifications to ensure the included studies were in line with the study aim. The final inclusion and exclusion criteria are presented in Table 2. The final searches were conducted between January 20, 2025 and January 31, 2025 (all database searches are shown in Attachment I).

**TABLE 2:** Inclusion and exclusion criteria based on the PEO framework.

PEO framework	Inclusion	Exclusion
Population: Nurses in hospital settings	Postgraduate/registered nurses Nurses with internationally comparable basic education Only hospital settings General wards/emergency departments	5 Specialist nurses, as they have another educational basis, meaning these nurses are not comparable with basic trained nurses 6 Specialist wards/units that require specialist nurses only 7 Studies where results cannot be separated between included professions 8 Studies are unclear about educational level of nurses
Exposure: Nurses' unclear responsibilities	Unclarity related to nurse's: 9 Professional/legal/disciplinary responsibilities 10 Responsibilities in the healthcare team 11 Organisational rearrangements 12 Extended responsibilities 13 Delegated responsibilities	14 Unclarity in other professions' responsibilities
Outcome: Influence on the nurses' role development	Influences on the nurse's: 15 Growth/learning development/needs 16 Role development in the team 17 Role development towards patients 18 Expanded role 19 Changed role 20 Adjusted role 21 Individual development	22 Influence on other professions' role development
Study type	23 Only empirical/primary research 24 Peer‐reviewed studies	25 Grey literature 26 Theoretical studies 27 Reviews
Language	28 English because of the risk of time‐consuming translations	
Time period	29 Limitation of 10 years (2014–2025) as the scope is related to modern hospital care	
Quality appraisal	30 Only ethically approved studies	31 Predatory journals

#### 3.2.2 Stage 3—Study Selection

When reading the titles and abstracts, studies were excluded from the search results according to the reasons noted in Table 2. In line with Arksey and O'Malley (2003), no formal quality appraisal of the included studies was conducted, as the aim was to map the existing research rather than assess the methodological rigour of the included studies. The initial screening meant including all studies that could be of interest for the study and excluding studies that did not appear to have relevance for the research question. Duplicates were removed manually during the screening process.

The reading of titles and abstracts was performed by the first author (MB). A sample of studies was double screened by the whole research team to ensure consistency in the application of inclusion criteria.

**FIGURE 1:**
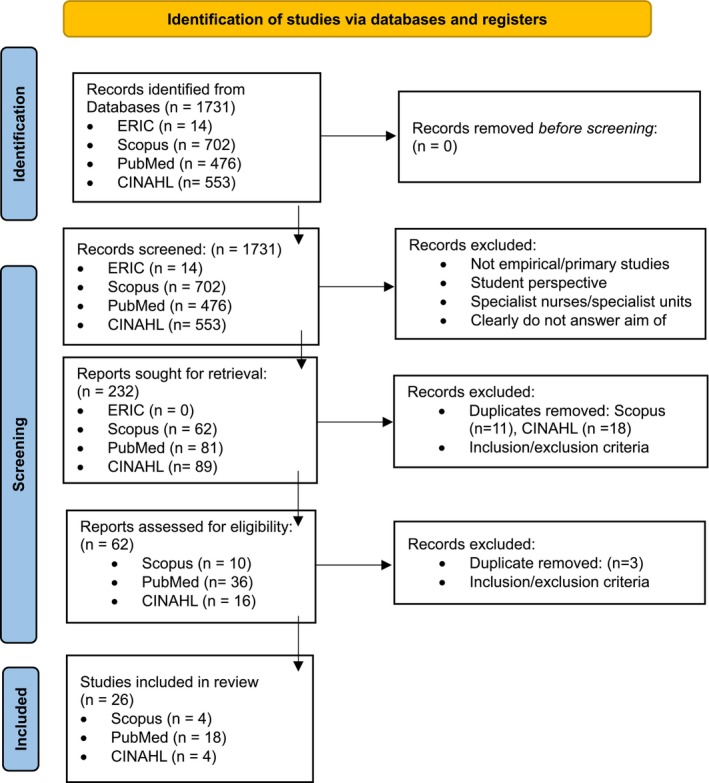
PRISMA flow chart over included studies. *Source:* Page et al. (2021).

The inclusion and exclusion criteria became the guide when including the final studies. The full text studies were downloaded to assess their relevance for the review. The studies were assessed based on the inclusion/exclusion criteria. All decisions were documented and charted (see Attachment I) for transparency, but also for the final display of all included studies (Sandelowski 2008; Sandelowski and Barroso 2007; Arksey and O'Malley 2003). All the studies that we were unclear about whether to include were saved for a second opinion to reach consensus for the final inclusion by the entire research team. The PRISMA flow chart (Page et al. 2021) was then used to finalise the display of the included and excluded studies (see Figure 1). A total of 26 studies were included, conducted between 2016 and 2024, from 15 countries; Nordic and European countries were most represented. All included studies are presented in Attachments I and III.

#### 3.2.3 Stage 4—Extracting and Charting the Data

As a first part of the analysis process, the data was extracted, organised and charted by MB and checked by all authors to ensure consistency (Arksey and O'Malley 2003). Data charting when doing a scoping review can be done in different ways; however, in this review, the extracted data included the studies' participants, setting, data design and outcomes. Studies with quantitative designs were narratively interpreted by summarising their findings in a narrative format, which enabled the comparison between different methodologies/designs (see Attachment II).

#### 3.2.4 Stage 5—Collating, Summarising and Reporting the Results

The analysis was done as a narrative interpretation of the studies, using a thematic analysis guided by the six steps described by Braun and Clarke (2006), which are listed below.

1. Familiarising with the data. The included studies were read in full text, and the results were specifically read several times to do a more active reading. Already in this stage, some patterns and ideas could be discerned, which were documented during the reading.
2. Generate initial codes. Here, codes were identified from the data, which meant systematically identifying interesting parts of the data and organising these parts into meaningful units to identify segments and codes in the data. This was done in Microsoft Word by MB, who summarised all the included studies in a table. The table was organised after the PEO framework, where meaningful segments from each study were summarised in separate columns for the ‘E’ and ‘O’. This organisation of data enabled the identification of codes from each segment, which were marked in different colours.
3. Identify/search for themes. In this step, the codes were grouped together into themes, where relations between codes and patterns were identified. The themes were developed on a broader, latent level than the initial codes, where the researchers sought to identify the underlying meaning and patterns in data (see Table 3).
4. Reviewing themes. Here, the themes were reviewed and revised several times. This was a back‐and‐forth process, where the initial main themes and subthemes were reorganised and analysed several times by the whole research team until consensus was reached. The themes were also assessed to the study aim and all themes were interpreted similarly according to a level of abstraction. Also, the accuracy of the text in every theme was assessed, where some texts were moved and/or removed.
5. Define and name themes (see Table 4 in the results section). Again, the text was read with accuracy to its existing theme, and a summary of the essence of every theme was developed.
6. Producing the report. The results have been presented in a structured manner, where the included studies were separated into main/subthemes in a table by MB (see Attachment III).

**TABLE 3:** Example of codes to theme.

Extracted codes for nurses' unclear responsibilities	Extracted codes concerning the influence on the nurses' role development	Main theme
Extend the responsibility when the system fails When the responsibility is unclear, the nurses take on the responsibilities Nurses act as a quality assurance/cover system error by taking responsibility	Develop new nursing roles Develop a quality assurance role Extends the nursing role to cover system deficits and unclarities	Evolving the nursing role through adaptability, leadership and personal engagement

## 4 Results

The thematic analysis resulted in two main themes—‘Navigating role ambiguity through trust and a learning community’ and ‘Evolving the nurses*’ role through adaptability, leadership and personal engagement*’*—*which will be elaborated upon below (see Table 4).

**TABLE 4:** Final themes and subthemes.

Main theme	Subtheme
Navigating role ambiguity through trust and a learning community	Role uncertainty contributed to mistrust of competence Managing an expanded role as a nurse Strengthening the nurses' role in a supportive environment
Evolving the nurses' role through adaptability, leadership and personal engagement	Professional growth by personal engagement and adaptive traits Shifting towards supportive leadership and coordination

### 4.1 Navigating Role Ambiguity Through Trust and a Learning Community

This theme describes how the nurses' responsibilities and the following nurses' roles in the healthcare teams were often unclear and subject to change. The included studies highlighted how nurses often assumed additional duties due to high expectations from both society and healthcare organisations, sometimes compensating for system deficits. The nurses took on additional responsibilities in patient care, ward coordination and even medical decision‐making, often without formal training. This ambiguity could lead to overlapping responsibilities with other professionals, causing uncertainty in teamwork and patient care. Here, trust in one's own competence and that of colleagues was essential for collaboration and patient safety. Learning in a safe community through clear role definitions, mutual respect and structured communication could enhance professional collaboration, improve patient safety and support nurses in developing and strengthening their roles in the team.

#### 4.1.1 Role Uncertainty Contributed to Mistrust of Competence

The uncertainty of the professional roles in the team could contribute to distrust between the professionals (Henshall et al. 2018). The distrust and uncertainty of competence were something that the nurses were facing towards the assistant nurses (Willman et al. 2021). As the autonomy of the assistant nurses' role also was unclear, they could make their own decisions without necessary nursing supervision. This could lead to the nurses mistrusting the assistant nurses' competence and responsibility to fulfil the delegated tasks (Henshall et al. 2018; Carroll et al. 2024). Hence, being able to trust each other's competence was significant for the nurses to coordinate the work and to trust tasks being fulfilled without having to double‐check the assistant nurses' actions (Carroll et al. 2024). Since the nurses understood that some assistant nurses lacked the necessary skills in patient care, being aware of these limitations was crucial for patient safety (Willman et al. 2021). However, the trust could be restricted towards specific individuals among the assistant nurses, which is why the nurses needed to have personal knowledge about the assistant nurses in their team (Carroll et al. 2024). Furthermore, trusting the assistant nurses to know when to report patient deterioration and vital signs to the nurses was essential for the nurses' ability to have control over the situation (Willman et al. 2021). The nurses were assumed to have the overall responsibility for patient care, which is why the assistant nurses did not participate in rounds or verbal reports and thus lacked proper information about the patients (Chua et al. 2022). Consequently, the unclarified roles between nurses and assistant nurses made it unclear who was responsible for mistakes in patient care, or who was responsible when assistant nurses missed patients' deterioration (Enggaard et al. 2024). To clarify who was responsible for the patient during patient handovers, improved and structured information between the professionals and working shifts was essential (Dúason et al. 2021).

#### 4.1.2 Managing an Expanded Role as a Nurse

The nurses experienced high expectations from both society and the healthcare organisation to expand their role and take on more responsibility when the healthcare organisation required it. The nurses could experience being respected by colleagues and patients when advancing their competences and skills (Bolme et al. 2021). It was difficult to accept and fulfil these expectations (Trettin et al. 2024). Taking on more responsibility also led to feelings of being on shaky ground, with fear and lack of competence for the extended responsibilities where the nurses learned along the way (Trettin et al. 2024; Bolme et al. 2021). The nurses felt that they were expected to manage their extended responsibilities competently but could experience being distrusted in the team concerning their level of competence (Liang et al. 2021). Also, as Agerholm et al. (2023) found, the nurses could take on more responsibilities when they experienced the healthcare system fail. The nurses took on responsibilities they felt outspokenly obliged to take, but the nurses also compensated for system deficits and errors by acting as quality assurance for patient care when routines and guidelines were insufficient to meet patients' needs (Agerholm et al. 2023). Dúason et al. (2021) described how nurses were quicker to take on the role of patient responsible when patients arrived at the emergency department from the ambulance, compared to the physicians (Dúason et al. 2021). In addition, the nurses' engagement and role perception influenced their patient relationships, workload and tasks. Hence, the responsibility and knowledge development relied on the individual nurses, who often went beyond their role as nurses to support and take responsibility for the patients (Bafandeh Zendeh et al. 2022).

Logan et al. (2021) discussed how the changing roles among the nurses, physicians and pharmacists had led to nurses performing tasks and taking on responsibilities that they did not have competence in, such as expanded medical responsibilities. The nurses felt that their roles as nurses could be expanded and that they were qualified to take on a greater responsibility for monitoring patients and handling prescriptions for patients when needed (Logan et al. 2021). Similarly, Wuyts et al. (2022) found that, depending on working shifts, the nurses could be taking over medical responsibilities they did not have clear medical prescriptions or competence to perform. For example, during night shifts or acute situations, the nurses needed to assess lab results and make medical decisions based on the wards' routines and guidelines, while missing medical prescriptions from the physician (Wuyts et al. 2022). Further, nurses had also expanded their roles into ward coordinators and took responsibility for monitoring the wards' bed occupancy, a role normally performed by the ward manager. However, this was a complex role that the nurses needed support from the assistant nurses to manage, a support that the nurses later declined from the assistant nurses to claim the ward coordinating role as their own (Van Schothorst‐van Roekel et al. 2021).

To manage the uncertainty when taking on more responsibilities, the nurses wished for more education to manage the uncertainty. Even if the pharmacists were assessed to possess better competence for drug management, the nurses saw them as a threat and feared being exchanged for pharmacists at the ward (Logan et al. 2021). Sjölander et al. (2017) had similar findings when describing that the purpose of introducing pharmacists at the ward was to improve drug management and to save time by assisting the nurses, although the role uncertainty caused concerns among the nurses about being replaced by pharmacists (Sjölander et al. 2017).

#### 4.1.3 Strengthening the Nurses' Role in a Supportive Environment

The nurses' responsibilities could be rapidly reorganised due to staffing shortages and organisational changes, which could cause the nurses' role to overlap with those of other professionals in the team and cause uncertainty between the professionals and towards the patients (Woldring et al. 2023). Jin et al. (2024) described the nurses' role in the healthcare team as challenging due to unclear responsibilities and duties that did not align with the expectations nor the responsibilities of the nursing profession. Thus, strengthening and clarifying the nurses' role was stressed as crucial to optimise and contribute to the teamwork (Jin et al. 2024).

When the nurses' role was uncertain, the teamwork and healthcare community could help strengthen the nurses' role (Milton et al. 2022; Jin et al. 2024) as the nurses could learn from mistakes in a safe environment without fear of being blamed if something went wrong (Espinoza et al. 2016; Dúason et al. 2021). Close teamwork could also decrease the risk for hierarchical orders between the nurses and physicians, as professional barriers and strict routines could be a risk for patient safety (Mink et al. 2023). A strong and safe community was characterised by a feeling of togetherness and belonging, including not being afraid of reprimands (Mink et al. 2023; Milton et al. 2022). When working closely together in the team, the teamwork enabled continuous learning for the nurses when taking on new assignments (Bafandeh Zendeh et al. 2022). Also, the nurses were able to develop their responsibilities by learning from other professionals in their daily work (Bolme et al. 2021). Reflecting on and debriefing patient situations also contributed to better teamwork (Milton et al. 2022). This kind of collaboration has been said to contribute to nurses' increased confidence, self‐efficacy, self‐directed learning and interprofessional learning (Mink et al. 2023).

Building a close and safe teamwork required clarified goals, roles and a shared decision‐making process where the nurses' roles were strengthened and clarified in the team (Plantinga et al. 2024). Carroll et al. (2024) and Milton et al. (2022) felt similarly, emphasising the importance of a team and all professionals being dependent on each other (Carroll et al. 2024; Milton et al. 2022). The interdependency between the professionals required mutual respect, communicated expectations of each other's performance and fulfilment of tasks. There was also a need for clear organisation, planning and leadership to avoid power confusion between the professionals (Carroll et al. 2024). These results are in line with those of Timmins et al. (2018), who argued for the importance of communication and collaboration for sharing responsibility on all organisational levels within a healthcare system (Timmins et al. 2018), a result that was also supported by Logan et al. (2021).

### 4.2 Evolving the Nurses' Role Through Adaptability, Leadership and Personal Engagement

This theme highlights how nurses' personal traits, motivation and adaptability played a central part in how the nurses embraced the expanded responsibilities in the changing healthcare environment. The nurses demonstrated a strong drive for professional development, leading them to take on responsibilities beyond their formal roles as nurses to ensure safe care and support others in the team. The nurses' own interpretation of their roles affected their workload, their engagement and their collaboration in the team. Hence, our results show that the nurses' roles were largely determined by their adaptive traits and their abilities and skills to coordinate and lead their own and others' work in relation to the organisational prerequisites.

#### 4.2.1 Professional Growth by Personal Engagement and Adaptive Traits

Trettin et al. (2024) found that expanding nurses' roles when needed was something the nurses felt pride in. The nurses were driven by their motivation to learn and to increase their knowledge. The nurses were willing to take on more responsibilities to be useful, which could mean entering another ward specialty and learning new skills when needed. The nurses were characterised by a will and desire to go beyond what was expected of them as nurses (Agerholm et al. 2023; Bafandeh Zendeh et al. 2022). Liang et al. (2021) also identified personal traits that were valuable in a changing healthcare environment, such as the nurses having the ability to quickly respond to changing organisational prerequisites and being adaptable, inventive and able to improvise (Liang et al. 2021). Other valuable traits were the nurses' sense of humour (Liang et al. 2021) and ability to face challenges positively and as opportunities for personal and professional development (Bolme et al. 2021). Furthermore, clarifying the leadership responsibility through work organisation and planning was valuable for the nurses' roles development in relation to other professionals, especially assistant nurses (Carroll et al. 2024).

The nurses' engagement and how they interpreted their role as a nurse also affected their workload, tasks and duties. The nurses' engagement affected their relationship with the patients and colleagues, where their competence to be a role model for both patients and colleagues required updated knowledge and clinical experience (Agerholm et al. 2023). Here, collaboration and communication with other professionals and the prerequisites to develop their own expertise for the extended role as a nurse became significant (Enger and Andershed 2018). Hence, the nurses took on great personal responsibility to acquire the competence needed for their role and duties (Agerholm et al. 2023). The nurses felt that the expanded tasks sharpened them and supported their professional development (Bolme et al. 2021). However, developing adequate competence to take on responsibilities beyond their own competence could be hindered by time, clinical experience and resources at the wards. Also, the unclear limits of the nurses' responsibilities could affect their ability to get adequate training and skills for the work they were performing (Jin et al. 2024). The nurses believed that competence and skills could not be learned or achieved from books but were gained from clinical experience and tacit knowledge (Trettin et al. 2024). Here, written guidelines and assessment tools were believed to hinder the nurses' development of clinical assessment skills and posed a risk of neglecting their responsibility to observe and assess patients in bedside care (Jensen et al. 2019). On the other hand, the nurses could use assessment tools as supplementary guidance for being able to take responsibility for their own interpretations and assessments of the patients' overall status, before calling a physician (Vitale et al. 2024).

#### 4.2.2 Shifting Towards Supportive Leadership and Coordination

Enggaard et al. (2024) found that the nurses also took on a coordinating and supportive role towards the assistant nurses, such as preparing them for tasks and making them aware of special attention needed in patient care. Hence, to uphold patient safety, the nurses adjusted their roles to the situation and took responsibility for other team members' work (Enggaard et al. 2024). Hence, working closely together also enhanced the nurses' awareness of their own and others' competence, to better coordinate their own and others' work, which also developed their communication and delegation skills in the team (Milton et al. 2022). The nurses needed to be adjustable to the healthcare environment and current workload (Liang et al. 2021). However, the role expectations of the nurses could differ according to the healthcare setting (Jensen et al. 2019). In surgery care, the nurses needed to adjust between stepping into the role of an assistant to the physician and taking on a more autonomous role in post‐operative care, where the nurses' holistic perspective of patient care was needed and recognised as valuable (Espinoza et al. 2016).

The nurses could have their own expectations of other professionals in the team and delegate tasks and responsibilities when considering this to be appropriate (Enggaard et al. 2024). Controls of vital signs were typically activities that could be delegated to assistant nurses (Chua et al. 2022). However, assessing the results of vital signs was beyond the competence of the assistant nurses and required the nurses to have a supportive and teaching role towards the assistant nurses (Chua et al. 2022). Hence, delegating tasks when appropriate training and competence were lacking could be both time‐consuming and pose a risk for patient safety (Enggaard et al. 2024). Henshall et al. (2018) discussed the complexity of delegating nursing assignments from nurses to assistant nurses. It could be difficult for the nurses to revoke such delegation because the nurses did not want the assistant nurses to feel undervalued, and it could also lead to tensions between the professionals (Henshall et al. 2018). In addition, as the nurses assessed the patients' overall status, and not just the measurement results, the nurses felt that the assistant nurses were not competent enough to take on this responsibility (Langkjaer et al. 2023). Determining whether tasks could be delegated could be context‐ and situational‐dependent, but also dependent on the individuals' competence working in the team (Henshall et al. 2018). Here, the nurses' teamwork abilities, communication skills and delegation skills, and education to assistant nurses could be determined to provide safe patient care (Chua et al. 2022).

## 5 Discussion

This scoping review aimed to examine how unclear responsibilities influence nurses' professional role development in hospital settings. The findings indicate that nurses often face shifting and undefined responsibilities in daily work. The nurses frequently took on additional duties to meet high expectations and compensate for organisational shortcomings. These additional responsibilities could include coordinating and leading care and assuming duties beyond their formal role, often without adequate training. The nurses' ability to be adaptable, being motivated for professional growth and possessing a high sense of responsibility for patient safety were key factors that shaped their evolving roles. Furthermore, the nurses' confidence and self‐directed learning affected their professional role development. Trust, collaborative learning and structured communication appeared essential for the development and strengthening of the nurses' role within interdisciplinary teams.

### 5.1 Being the Adaptive Chameleon to Cover System Deficiencies

The results confirm findings from previous research that the nursing role needs to be adjusted to the quickly changing environmental changes in hospital care (Grover and Fritz 2022; Kagonya et al. 2023). Meaning, rearranging professional roles and the retraining of nurses' skills might be necessary for the development of nurses' necessary competencies in the future, as previously noted by Notarnicola et al. (2025) and Alshammari et al. (2024). The results show an evolution of the nursing role as being an adaptive chameleon, where the nurses needed to adjust the role and understand what responsibilities needed to be taken in each clinical situation. Here, the nurses necessitated the ability to have a holistic approach to patients' needs, seeing system deficits and having the skills to support, lead and educate colleagues. This result demonstrates a complexity in the nurses' ability to assess and learn in clinical situations. Having the ability to assess and adjust to clinical situations requires a nurse to be competent and experienced enough to grasp and comprehend the whole clinical situation, an ability that novice nurses are lacking, according to Benner (2001). Thus, as modern hospital settings are employing newly graduated nurses to a greater extent, the assumption is that a majority of nurses are lacking these adjustable abilities.

The results further showed how the nurses took on additional responsibilities based on their assessment of colleagues' competencies and the organisations' shortages to act as a quality assurance for patient care. However, due to the complex healthcare environment, this could be seen as a patient risk, as the results in this study demonstrate that the nurses feared not having enough competence for the extended responsibilities. Krautsched explained that nurses are accountable to individuals they care for, towards society and the profession by ensuring patient safety and upholding professional integrity (Krautscheid 2014). As also noted by Baker et al. (2020), this reasoning indicates that being able to adjust the nurses' role based on the needs of organisations, patients and colleagues will require a retraining of nurses, both during basic education and in clinical practice. Hence, as Notarnicola et al. (2025) also argued, the current educational preparation of nurses will need to be adjusted as well to better prepare nurses for the comprehensive and implicit responsibilities of being an organisational leader in hospital settings. Here, education and clinical practice need to be more adaptable and integrated after contemporary needs of nurses' competence.

### 5.2 Reliance on Nurses' Individual Motivation for Role Extension

This result demonstrates that the modern nurses' responsibilities are, to a greater extent, dependent on the individual nurses' personal desire and motivation to be accountable, which does not necessarily include support or organisational structures from the healthcare management. Personal motivational traits could be an asset in healthcare, as they could enhance performance and achievements (Credé 2018). However, such traits are often related to an individual's perception of receiving positive outcomes from their achievements (Credé 2018). In the present study, the motivation for nurses to go beyond their responsibilities was related to the will and desire to provide safe care, a will for professional growth, and wanting to be needed and irreplaceable. However, even though the concept of accountability provides a broader term for nurses' responsibilities, as described by Krautscheid (2014), without organisational structures and support, this term could be interpreted and fulfilled based on the individual nurses' personal ambitions. This means that when the nurses' responsibilities could be individually interpreted, the quality of care might also be determined by the individual nurse. This result indicates that individual accountability could be seen as risky, a result that Fawcett (2021) argued against, saying that the team‐based nursing model could sacrifice the individual accountability of nurses. Hence, these research results suggest that individual accountability could both be a risk and an asset in patient care.

### 5.3 Implicit Leadership Based on Nursing Accountability

The results further showed that individual accountability extended to the assistant nurses, as the delegation of tasks and responsibilities was based on which individuals the nurses trusted to carry out bedside care. Hughes et al. (2017) and Wagner (2018) stated that when nurses understand they remain accountable for nursing assignments, even if the tasks are delegated to assistant nurses, the nurses will more likely supervise the delegated tasks. Thus, the findings indicate that the expansion or delegation of professional responsibilities demands advanced assessment competencies, wherein the nurse's role entailed a sense of accountability for the overall functioning of the ward. This finding was previously discussed by Crevacore et al. (2023), who stated that nurses lacked the necessary leadership skills and education to ensure safe delegation, skills that become increasingly important as the number of assistant nurses grows in healthcare settings (Crevacore et al. 2023). The nurses could see potential risks of assistant nurses taking on extended nursing responsibilities and, similarly, the physicians could see the risks of nurses taking on extended medical responsibilities. Hence, the profession that ‘owned’ the responsibility defined for its profession could see the risks and need for competence and training to perform the tasks. Claiming ownership of tasks despite uncertainty in competence seemed to derive from an uncertainty about each profession's role in the team, where all professions needed to prove their value and contribution to the team. Consequently, the uncertainty of the different roles in the team created a need to make oneself indispensable, to be seen as valuable in the team and to hold on to the additional responsibilities. This could be seen as a risky development, as the profession holding on to a responsibility could be doing so for the wrong reasons. Here, communication and understanding the interdependency between the professionals is essential for interprofessional collaboration and to avoid overlapping and unclear responsibilities in the team. This result strengthens the argument that rearrangements of professional responsibilities require changes and clarifications on all organisational levels (Niezen and Mathijssen 2014), and a need for a clearer and more structured organisation in the healthcare teams (National Health Care Competence Council 2024).

The above reasoning suggests that the modern role of nurses should be redefined, as it does not exclusively include being nursing responsible, but implicit responsible as an organising and accountable leader. To support these changes, and to ensure patient safety, healthcare organisations must implement systemic changes, including clear delegation structures, opportunities for professional development and leadership training for nurses. The study has revealed that nursing accountability and the ensuing concept confusion about the role descriptions and responsibilities within healthcare teams need further exploration to explain how to bridge the basic education of nurses with contemporary hospital organisations' needs and challenges.

### 5.4 Implications for Nursing Practice

Individual nurses' willingness to expand their responsibilities depends largely on personal motivation and adaptability. Hospital organisations should recognise and encourage these traits to value the professional growth of nurses. At present, role expansion seems to be driven by individual nurses rather than being organisationally supported. Nursing accountability should be integrated into clinical practice by moving beyond the task‐based definition of nurses' responsibility. Nurses should be encouraged and trained to reflect on their clinical decisions and actions and be supported in their need for lifelong learning. A safe and supportive team environment was seen as crucial for nurses' daily work and learning, without fear of making mistakes. Nurse managers should prioritise the building of psychologically safe hospital environments where feedback is constructive, errors are seen as learning opportunities, and collaboration between professionals is encouraged. Further, the hospital organisations need to clarify the role expectations of all professions in the healthcare teams to avoid tensions and blurred professional boundaries. Hospital organisations should also provide clearer structures and guidelines for the safe delegation of tasks, recognising that the delegation of assignments in patient care requires guidelines and professional learning, and should not be based on the daily assessment of individual competencies. Training on how to safely delegate and lead daily work should represent a larger part of nurses' education and training in clinical practice.

Finally, the responsibilities of nurses should be redefined to reflect their evolving role as visible rather than hidden leaders. In this context, tacit knowledge and accountability should be recognised as integral to the contemporary leadership role of nurses. To ensure patient safety and maintain professional integrity, healthcare organisations need to actively accept and support the nurses through systematic leadership training which is a prerequisite for professional role development.

## 6 Conclusion

This review shows that unclear and expanding responsibilities are reshaping nurses' roles in modern hospital care. This changing nursing role was often driven by organisational shortcomings, high expectations regarding nursing performance and nursing responsibilities, and personal motivation among nurses. While nurses increasingly took on implicit leadership roles, coordination roles and extended responsibilities, this increased the risks regarding healthcare quality and patient safety when there was over‐dependence on nurses' individual traits and clinical assessments. To support safe healthcare and professional development of nurses' roles, nursing education and organisational structures need to better align with the evolving competencies that modern nurses require. This is done by clarifying the nurses' professional roles and strengthening interdisciplinary collaboration in the healthcare teams, including the role of leadership from personal drive to organisation‐driven leadership. However, more clinical research is needed to fully grasp the nurses' evolving roles and enable further recommendations for how to support nurses in clinical practice and prepare them during education.

### 6.1 Strengths and Limitations

This scoping review's trustworthiness is enhanced by its comprehensive searches in four databases and screenings of articles. Thus, the results of this review are supported by an extensive body of research. By including 26 articles in this review, the study also provides important insights into research gaps and existing evidence within the area. Doing a pilot search before doing the actual searches was also considered a strength of the study's trustworthiness, as the search blocks and search terms were tested and revised until reaching the most appropriate results. To manage the possibility of preunderstanding and individual interpretation during the review process, and to minimise the risk of bias, the entire research team was involved in all steps.

Transparency and replicability are both strengthened by the systematic and structural methodological approach used in the review. Using Arksey and O'Malley (2003) as a methodological framework enabled the review to be performed in this systematic and structured way. Reporting the methodological process in a clear way enables the replication of the study and enhances the research credibility as the process is transparent. Also, using the analysis framework for thematic analysis by Braun and Clarke (2006) contributed to the structure and systemic analysis, which improved the transparency and replicability of the study.

The review process could also reveal some limitations, which were managed as follows. Firstly, the inclusion and exclusion criteria were challenging to assess in some studies. This is because the definition of both professional roles and levels of education differs internationally, which is why it was more challenging to determine whether some studies should be included in the review. To be as certain as possible that the included nurses in the studies were comparable in terms of title and level of education, we excluded studies that we were uncertain about.

The fact that Nordic and European countries were overrepresented in the included studies should be taken into consideration when interpreting the results. The results from the included countries did not reveal any distinct differences in the nursing role that could be related to the region in which the study was conducted. One reflection made by the authors was that the Nordic studies more often mentioned how nurses were taking on additional responsibilities and were quicker to take on personal responsibility and additional tasks to cover system deficits. Also, the delegation of tasks, and unclarity of responsibilities between nurses and assistant nurses were more frequently discussed in the Nordic and European studies. Also, the title and role of assistant nurses differed across countries. Therefore, all titles were compared and all titles that were assessed to be comparable with a Swedish assistant nurse were grouped as a similar role/title, which can be seen in Attachment II. However, as there is not yet an international standard for the title of ‘nurse’ or ‘assistant nurse’, the results of this review need to be evaluated with caution. Further, given that no formal quality appraisal of the included studies was conducted (Arksey and O'Malley 2003), the results should be interpreted with caution.

## Author Contributions

**Mia Björk:** conceptualisation; methodology; validation; investigation; data curation; writing – original draft; project administration. **Irene Eriksson:** validation; review and editing. **Anette Ekström‐Bergström:** validation; review and editing. **Viola Nyman:** conceptualisation; validation; review and editing.

## Conflicts of Interest

The authors declare no conflicts of interest.

## Supporting information


**Data S1:** jan70345‐sup‐0001‐DataS1.docx.

**Data S2:** jan70345‐sup‐0002‐DataS2.docx.

**Data S3:** jan70345‐sup‐0003‐DataS3.docx.

**Data S4:** jan70345‐sup‐0004‐DataS4.pdf.

## Data Availability

Data is available by request.
