# Supplementary material for: The Evolving Role of Nurses in Hospital Settings—A Scoping Review
Source: J Adv Nurs. 2025 Nov 11;82(8):7704–16. doi: 10.1111/jan.70345 (PMC13356416; doi:10.1111/jan.70345)
Supplement: Supplementary file 1 — Data S1: jan70345‐sup‐0001‐DataS1.docx. [file JAN-82-7704-s003.docx]

Attachment I: Database searches

| **SCOPUS** | | | | | | |
| --- | --- | --- | --- | --- | --- | --- |
| **Search blocks** | **#** | **Search terms** | **Hits in Scopus  2025-01-20** | **1^st^ save Title/ Abstract** | **2^nd^ saves**  **Full text reading** | **3^rd^ Saves** |
| **Population: Nurses** | #1 | Nurs* | 1,048,613 |  |  |  |
| **Population: Hospital setting** | #2 | TITLE-ABS-KEY "in-patient" | 2,611,755 |  |  |  |
|  | #3 | TITLE-ABS-KEY "in patient care" | 11,065 |  |  |  |
|  | #4 | TITLE-ABS-KEY ward* | 274,399 |  |  |  |
|  | #5 | TITLE-ABS-KEY hospital* | 3,135,523 |  |  |  |
|  | #6 | TITLE-ABS-KEY "acute setting" | 4,164 |  |  |  |
|  | #7 | #2-#6 Boolean operator “OR” | 5,393,910 |  |  |  |
| **Exposure:  Nurses´ unclear responsibilities** | #8 | TITLE-ABS-KEY accountability | 90,560 |  |  |  |
|  | #9 | TITLE-ABS-KEY responsibility | 392,129 |  |  |  |
|  | #10 | TITLE-ABS-KEY obligation* | 93,216 |  |  |  |
|  | #11 | TITLE-ABS-KEY integrity | 457,686 |  |  |  |
|  | #12 | TITLE-ABS-KEY duty OR duties | 190,492 |  |  |  |
|  | #13 | TITLE-ABS-KEY liabilit* | 99,525 |  |  |  |
|  | #14 | #7-#12 Boolean operator “OR” | 1,254,390 |  |  |  |
| **Outcome:  Influence on the nurses´ role development** | #15 | TITLE-ABS-KEY "professional development" | 76,302 |  |  |  |
|  | #16 | TITLE-ABS-KEY "professional role" | 23,066 |  |  |  |
|  | #17 | TITLE-ABS-KEY "professional growth" | 4,800 |  |  |  |
|  | #18 | TITLE-ABS-KEY "skill development" | 17,900 |  |  |  |
|  | #19 | competenc* | 495,398 |  |  |  |
|  | #21 | #16-#19 Boolean operator “OR” | 596,009 |  |  |  |
| **Limitations** | #23 | 2014-2025  Articles  English  Peer-reviewed or similar database limitation |  |  |  |  |
| **Combined search blocks** | #22 | #1 AND #7 AND #14 AND #21 AND #23 | 702 | 62 | 10 (+11 duplicates) | 4 |
| Reason exclusion:  Duplicates, work satisfaction/performance, continuous learning, new nurses´ transition/learning, spec nurses, spec units/settings, nurses in leader positions, reviews, predatory journals | | | | | | |

| **EBSCO ERIC** | | | | | | |
| --- | --- | --- | --- | --- | --- | --- |
| **Search blocks** | **#** | **Search terms** | **Hits in ERIC 2025-01-30** | **1^st^ save Title/ Abstract** | **2^nd^ saves**  **Full text reading** | **3^rd^ Saves** |
| **Population: Nurses** | #1 | Nurs* | 17,479 |  |  |  |
| **Population: Hospital setting** | #2 | "In-patient" | 15,977 |  |  |  |
|  | #3 | Inpatient | 1,266 |  |  |  |
|  | #4 | "In patient care" | 1,188 |  |  |  |
|  | #5 | Ward* | 5,679 |  |  |  |
|  | #6 | Hospital* | 12,090 |  |  |  |
|  | #7 | "Acute setting" | 5 |  |  |  |
|  | #8 | #2-#7 Boolean operator “OR” | 25,565 |  |  |  |
| **Exposure:  Nurses´ unclear responsibilities** | #9 | Accountab* | 2 |  |  |  |
|  | #10 | Responsib* | 71,761 |  |  |  |
|  | #11 | Obligation* | 5,264 |  |  |  |
|  | #12 | Integrity | 6,876 |  |  |  |
|  | #13 | Duty | 8,904 |  |  |  |
|  | #14 | Liabilit* | 3,002 |  |  |  |
|  | #15 | #8-#13 Boolean operator “OR” | 89,809 |  |  |  |
| **Outcome:  Influence on the nurses´ role development** | #16 | "Professional development" | 61,401 |  |  |  |
|  | #17 | "Professional role" | 775 |  |  |  |
|  | #18 | "Professional growth" | 3,559 |  |  |  |
|  | #19 | "Skill development" | 36,248 |  |  |  |
|  | #20 | competenc* | 112,376 |  |  |  |
|  | #21 | Learn* | 679,039 |  |  |  |
|  | #22 | #15-#20 Boolean operator “OR” | 574,526 |  |  |  |
| **Limitations** | #23 | 2014-2025  Academic journals  English  Peer-reviewed or similar database limitation |  |  |  |  |
| **Combined search blocks** | #24 | #1 AND #8 AND #15 AND #22 AND #23 | 14 | 0 | - | - |
| Reason exclusion:  Only nurse students and educational environment | | | | | | |

| **PubMed** | | | | | | |
| --- | --- | --- | --- | --- | --- | --- |
| **Search blocks** | **#** | **Search terms** | **Hits in PubMed 2025-01-31** | **1^st^ save Title/ Abstract** | **2^nd^ saves**  **Full text reading** | **3^rd^ Saves** |
| **Population: Nurses** | #1 | Title/AB Nurs* | 573,068 |  |  |  |
| **Population: Hospital setting** | #2 | Title/AB in-patient* | 2,2 million |  |  |  |
|  | #3 | Title/AB inpatient | 125,482 |  |  |  |
|  | #4 | Title/AB “in patient care” | 8,661 |  |  |  |
|  | #5 | Title/AB Ward* | 79,242 |  |  |  |
|  | #6 | Title/AB Hospital* | 1,8 million |  |  |  |
|  | #7 | "Acute setting" | 3,023 |  |  |  |
|  | #8 | #2-#7 Boolean operator “OR” | 3,8 million |  |  |  |
| **Exposure:  Nurses´ unclear responsibilities** | #9 | Title/AB Accountab* | 25,771 |  |  |  |
|  | #10 | Title/AB Responsib* | 649,809 |  |  |  |
|  | #11 | Title/AB Obligation* | 16,287 |  |  |  |
|  | #12 | Integrity | 186,582 |  |  |  |
|  | #13 | Duty | 29,208 |  |  |  |
|  | #14 | Liabilit* | 25,546 |  |  |  |
|  | #15 | #9-#14 Boolean operator “OR” | 914,147 |  |  |  |
| **Outcome:  Influence on the nurses´ role development** | #16 | "Professional development" | 16,122 |  |  |  |
|  | #17 | "Professional role" | 2,420 |  |  |  |
|  | #18 | "Professional growth" | 1,548 |  |  |  |
|  | #19 | "Skill development" | 3,235 |  |  |  |
|  | #20 | competenc* | 123,136 |  |  |  |
|  | #21 | Learn* | 737,604 |  |  |  |
|  | #22 | #16-#21 Boolean operator “OR” | 851,761 |  |  |  |
| **Limitations** | #23 | 2014-2025  Humans  English  Abstract  Full text  Peer-reviewed or similar database limitation |  |  |  |  |
| **Combined search blocks** | #24 | #1 AND #8 AND #15 AND #22 AND #23 | 476 | 81 | 36 | 18 |
| Reason exclusion: New nurses´ role transition, spec nurses, other spec roles, nurse leaders, students, not primary research, responsibility not associated with the nursing role, other settings, nurse practitioners, spec units | | | | | | |

| **CINAHL** | | | | | | |
| --- | --- | --- | --- | --- | --- | --- |
| **Search blocks** | **#** | **Search terms** | **Hits in CINAHL 2025-01-30** | **1^st^ save Title/ Abstract** | **2^nd^ saves**  **Full text reading** | **3^rd^ Saves** |
| **Population: Nurses** | #1 | AB Nurs* | 365,072 |  |  |  |
| **Population: Hospital setting** | #2 | AB in-patient* | 402,897 |  |  |  |
|  | #3 | AB inpatient | 54,054 |  |  |  |
|  | #4 | AB “in patient care” | 3,328 |  |  |  |
|  | #5 | AB Ward* | 28,794 |  |  |  |
|  | #6 | AB Hospital* | 477,874 |  |  |  |
|  | #7 | AB "Acute setting" | 984 |  |  |  |
|  | #8 | #2-#7 Boolean operator “OR” | 840,844 |  |  |  |
| **Exposure:  Nurses´ unclear responsibilities** | #9 | Accountab* | 11,453 |  |  |  |
|  | #10 | Responsib* | 86,686 |  |  |  |
|  | #11 | Obligation* | 6,302 |  |  |  |
|  | #12 | Integrity | 19,748 |  |  |  |
|  | #13 | Duty | 12,783 |  |  |  |
|  | #14 | Liabilit* | 4,648 |  |  |  |
|  | #15 | #9-#14 Boolean operator “OR” | 135,185 |  |  |  |
| **Outcome:  Influence on the nurses´ role development** | #16 | "Professional development" | 50,232 |  |  |  |
|  | #17 | "Professional role" | 51,286 |  |  |  |
|  | #18 | "Professional growth" | 1,318 |  |  |  |
|  | #19 | "Skill development" | 225 |  |  |  |
|  | #20 | competenc* | 130,343 |  |  |  |
|  | #21 | Learn* | 263,079 |  |  |  |
|  | #22 | #16-#21 Boolean operator “OR” | 219,605 |  |  |  |
| **Limitations** | #23 | 2014-2025  Academic journals  English  Peer-reviewed or similar database limitation |  |  |  |  |
| **Combined search blocks** | #24 | #1 AND #8 AND #15 AND #22 AND #23 | 553 | 89 | 16 (+18 duplicates in PubMed) | 4 |
| Reason exclusion: Duplicates, reviews, inclusion/exclusion criteria, spec nurses (ICU/NP/spec care/preceptors/advanced nurses), not empirical, nurse managers, role transition new nurses, not professional responsibility, poster presentations, continuous education/learning, learning strategies, students, other settings | | | | | | |
