# Supplementary material for: The Evolving Role of Nurses in Hospital Settings—A Scoping Review
Source: J Adv Nurs. 2025 Nov 11;82(8):7704–16. doi: 10.1111/jan.70345 (PMC13356416; doi:10.1111/jan.70345)
Supplement: Supplementary file 2 — Data S2: jan70345‐sup‐0002‐DataS2.docx. [file JAN-82-7704-s002.docx]

Attachment II: Data extraction and data charting

| Author (Year)  Study location | Study population | Design | Extracted data |
| --- | --- | --- | --- |
| Agerholm et al. (2023), Denmark/ Sweden | Nurses (n=12), hospital care | Qualitative, interviews | Hospital nurses were working beyond own professional duty/work due to strong feelings of responsibility for the patients in relation to the patients´ discharges. The nurses expanded own roles when the system failed or when routines, guidelines was inadequate to meet patients´ needs. The nurses needed to interpret information and conversations from other colleagues to provide to the patients. When responsibility was unclear, the nurses took the responsibility.  Nurses as the quality assurance in the healthcare system. Nurses covers system errors by the way they work and take on new roles and responsibilities |
| Bafandeh Zendeh et al. (2022), Iran | Nurses (n=18), hospital wards | Qualitative, interviews | Engagement and what the nurses interpret as their role, affects their relation to the patients and their workload and tasks. Responsibility was dependent on the individual nurse. Nurses went beyond their roles to provide support to their patients. The duty of the nurse and need for knowledge is the nurses´ own responsibilities. Increase knowledge and experience to be a role model for patients and colleagues. Nurses took a great responsibility to have the competence needed. |
| Bolme et al. (2021), Norway | Nurses (n=12), hospital care | Qualitative, interviews | Nurses were trained to take on physicians´ task. New tasks caused uncertainty but also respect among colleagues and patients. Felt proud with a greater level of responsibility, helped them to develop professionally, sharpened them, higher self-esteem. Made a difference and saving resources. Required good teamwork in the nursing team when lacking physician. A well-functioning nursing team was more efficient than physician collaboration. Social environment important (jokes, humor). Need for continuous learning when taking new tasks (theory/practice), need to answer questions from patients. Rather wish for nursing supervision than physician. |
| Carroll et al. (2024), UK | Nurses (n=33), acute hospital settings | Qualitative, non-participate observations and interviews | Role of nurses and assistant nurses*. They had different, individual tasks, but needed support from the other to complete the work. Personal characteristics affected the teamwork. Claiming each other as “their nurse/their assistant nurse”, isolated pair dyad. Assistant nurses acted non-dependent when carry on own tasks, required shared expectations and understanding. Nurses decided over time how/who to trust by initially double-checking assistant nurses´ work and take responsibility for the tasks being done. Interdependency. The need for clear leadership/planning/organization of work in the dyad. Managing risks of power structures and hierarchies in the dyad. Clarify the interdependency between nurses and assistant nurses. |
| Chua et al. (2022), Singapore | Nurses (n=11), general hospital wards | Qualitative, interviews | Nurses and assistant nurses* did not jointly receive patient information when starting their shift. Assistant nurses needed better information from the nurses to assess patient´s need of care. Role expectations of each other. Vague roles and responsibilities between nurses and assistant nurses. Expectations of responsibilities for e.g. vital signs. Assistant nurses task oriented. Did not report/could not assess patient´s abnormal vital signs. A need for collaboration and support between nurses and assistant nurses. Experienced assistant nurses were hindered to act due to hierarchies and education; they wanted more autonomy. The need for more/better communication, delegation, teamwork abilities, teaching to assistant nurses from the nurses |
| Dúason et al. (2021), Iceland | Nurses (n=7), emergency departments and ambulance | Qualitative, interviews | Unclear responsibilities according to legal/moral responsibility between professionals. The person responsible at patient handovers was the one with highest education: nurse/physician. This became unclear when level of experience differed. All professionals were accountable for own actions. Also unclear when responsibility shifts between ambulance/emergency dep. Nurses were quicker to take on patient responsibility than physicians. Skills varied between professions and individuals in the team, teamwork and collaboration was essential. Structured communication/handover tools like SBAR. Guidelines. The need for feedback in the team to learn and develop from mistakes. |
| Enger & Andershed (2018), Norway | Nurses (n=8), hospital ward care | Qualitative, open interviews | Nurses needs to take on an increased responsibility beyond own competence to care for ICU-patients on general wards. Limited resources, time and experience to take on the responsibility and challenge to take care of ICU patients. The need for communication, collaboration with ICU staff, good reporting skills, clinical gaze and expertise, increase knowledge and competence for critical ill patients. |
| Enggaard et al. (2024), Denmark | Nurses (n=16), hospital ward care | Qualitative, semi-structured interviews | Delegation of nursing tasks to assistant nurses* was complex. Nursing activities that matched the assistant nurses´ competences could be delegated, risk for fragmented care. Activities such as NEWS could be delegated which exceeded the assistant nurses´ competence. Could be time-consuming. Unclear who was responsible when assistant nurses made mistakes or did not report patient deterioration. Nurses took on responsibility to prepare assistant nurses for tasks and special attentions. Difficult to grasp the changed and undefined role. Challenged the nurses´ responsibility and role and hampered nurses´ own clinical work. Also, nurses had a coordination role and supportive role to the assistant nurses. Nurses took on responsibility for the whole team to uphold patient safety. |
| Espinoza et al. (2016), Chile | Nurses (n=11), perioperative care | Qualitative, in-depth/semi-structured interviews | Nurses felt responsible for the entire surgical process, but the role was framed by experience. Both feelings of being the one to blame if something goes wrong and being a part of a team to develop skills. Unclear of professionals´ roles in the surgery chain. Nurses´ role were clearer and more recognized and autonomous post op where a holistic, proactive perspective was needed. During surgery could feel like assistant. Adjustable role. |
| Henshall et al. (2018), UK | Nurses (n=7), hospital ward care | Qualitative, focus groups | Unclear roles between nurses and assistant nurses*. Assistant nurses took on extended roles and performed nurses´ work. Unclear division of responsibility, varied between settings. Nurses did not want the assistant nurses to feel undervalued and take skills away. Lack of nurses was one reason. Nurses were unsure of assistant nurses´ competence, and that assistant nurses could take more responsibility different days depending on nursing staffing and local manager decisions. Assistant nurses thought nurses was afraid they would take over their work and need to prove themselves to take on extended roles. Unclear autonomy of assistant nurses, some took own clinical decisions with/without nursing supervision.  A need for a role clarity where assistant nurses´ core competence need description on org level. The assistant nurses felt the nurses did the same work, only drugs were specific for nurses. Nurses was named as professionally accountable due to registered profession. Nurses felt responsible for the assistant nurses´ work. Tensions when nurses did not let assistant nurses take on nursing tasks/role. Distrust. Need for a role clarity on org level and role expectations. Change the role to Nurse Assistant to clarify. Ownership an excellence in patientcare. Value the different professions contribution to the team by clarification. |
| Jensen et al. (2019), Norway | Nurses (n=14), hospital ward care | Qualitative, in-depth/semi-structured interviews | Standardized tools as NEWS were experienced to undermine nurses´ clinical assessments and responsibilities. NEWS could be a risk that nurses miss out on their responsibilities to perform clinical observations and seeing the patients´ deterioration. Some did not feel NEWS changed their professional responsibilities. Risk of being locked to guidelines, nurses summoned the physician based on NEWS results, not their assessments, could be wrong. Others felt NEWS helped to assess patients’ status and when to act. NEWS helped nurses being confident in conversations and collaboration with physicians. Nurses felt professional accountability was beyond using tools such as NEWS. Nurses need to increase/use clinical judgement alongside with objective tools. Professional accountability is more than following guidelines. The community of practice, collaboration skills with the physician. |
| Jin et al. (2024), China | Nurses (n=14), general hospital care | Qualitative, in-depth semi-structured interviews | Nurses reported a mismatch between their actual work and their professional roles, often due to organizational challenges, unclear responsibilities, and staffing shortages. Their duties were dominated by routine tasks that failed to reflect the full scope of nursing expertise. The lack of standardized training, workflows, and role clarity prevented nurses from effectively fulfilling their professional responsibilities. This misalignment hindered their contribution to healthcare teams. Strengthening the nursing profession requires clearer role definitions, improved interprofessional collaboration, and enhanced, systematic training to address knowledge gaps and meet growing healthcare demands. |
| Langkjaer et al. (2023), Denmark | Nurses (n=32), hospital wards | Qualitative fieldwork study, interviews/ observations | Risks for information to be overlooked or missed when patient-assessments was done by other healthcare staff. Nurses did other assessments of patients´ overall status. The tools for assessment such as NEWS helps nurses take responsibility for the patients without having a physician at the ward. A responsibility that assistant nurses* could not take.  Assessment tools affected nurses´ professionalism and ability to determine when to signal concerns to the physician. The nurses could assess and modify the score using own competence before signal concerns (some nurses did no own interpretations) |
| Liang et al. (2021), Taiwan | Nurses (n=16), hospital ward/emergency care | Qualitative, interviews | Distrust between professionals´ competence in patient care. Nurses were expected to manage new tasks, training, required knowledge. Being adaptable, expected to do what was required.  Need to be adjustable to a rapid changing environment, abilities to improvise and be inventible to manage a pressured situation. Nurses needed to have ability to update skills and knowledge in response to changes to take responsibility of the care. Positive character traits such as humor when facing challenges. |
| Logan et al. (2021), Wales/UK | Nurses (n=220 and n=8), acute/ward care | Mixed method: survey/ interviews | Unclear responsibilities/roles between the nurses/physicians, physicians putting a lot of trust on nurses´ judgements on medication/follow prescriptions. Nurses thought this was physicians´ responsibilities. Nurses felt their roles could be extended and that they were qualified to more responsibility in monitoring patients. Physicians (and pharmacists) expressed nurses could be drawn into roles they do not have competence to perform and tasks such as prescription should not be extended to nurses. Nurses did not have the whole picture. Could be disaster.  Nurses were lacking drug knowledge. Collaboration skills were crucial with especially physicians and pharmacists. Collaboration was used before education. Nurses were performing roles that do not have competence to do (according to physicians). The need for education, multidisciplinary support, org structures for nurses being able to extend their role. |
| Milton et al. (2022), Sweden | Nurses (n=12), emergency department | Qualitative, semi-structured interviews | Realizing it was very difficult to be a nurse or a physician without the other profession. Fear of reprimands when suggesting treatment or care that violates professional boundaries. Tensions between roles and responsibilities. Supportive colleagues, teamwork, role-models. The need for organized reflections, debriefing for better collaboration, communication, self-awareness and professional motivation. |
| Mink et al. (2023), Germany | Nurses (n=4), hospital ward care | Qualitative, semi-structured interviews | A training-ward follow up study with nurses and physicians. Positive experience of blurring the boundaries between the professionals. Better collaboration, self-directed learning in bedside care and increased interprofessional learning where tasks were exchanged in bedside care. Rounds and teamwork positive for development. Need to reflect together in the team. Reflection on roles and responsibilities and less hierarchies = team. Working and learning together, also socially boundary breaking. Positive not having strict routines between the professions. Barriers, lacking communication and hierarchies was believed to risk patient-safety. Broaden own competence when working with others. Increase understanding of other professionals´ tasks. Improved communication skills and the use of communication tools such as SBAR. Increased confidence and self-efficacy. Broaden competence was positive. Better coordination skills and delegation skills (both own work and others). |
| Plantinga et a. (2024), Netherlands | Nurses (n=14), different hospital settings | Qualitative, individual interviews | Shared decision making (SDM) with patients, relatives, and healthcare providers. Clear goals for patient care were lacking. SDM was believed to be physician´s task. Nurses were lacking competence for SDM as they did not see this as their task. The nurses´ role in multidisciplinary SDM needed to be clarified to improve the nursing domain |
| Sjölander et al. (2017), Sweden | Nurses (=9), hospital ward care | Qualitative, semi-structured interviews | Positive experience of using pharmacists on the ward, as more competence in drugs, saving time, helping nurses. Other nurses are afraid being exchanged for pharmacists. Expectations of the different roles were unclear. Unclear professional roles, responsibilities and expectations in the team could be a barrier for introducing pharmacists on the ward. |
| Timmins et al. (2018), Haiti | Nurses (n=13), hospital ward care | Qualitative, observations and semi-structured interviews | Unclear roles in wound-care. Nurses claimed it was nursing responsibility, but the observations showed this was performed by medical residents as it is part of their training but considered it to be nursing responsibility bot thought nurses was not aware of this. The need for better collaboration with physicians for responsibility sharing. Nurses lacked clinical training to do the task. Inconsistency between education and clinical practice. Wished for wound care protocol and ongoing training sessions. |
| Trettin et al. (2024), Denmark | Nurses (n=12), infectious disease wards | Qualitative, focus groups | Feeling obligated and responsible as a nurse to take on new role during the pandemic. Demands from society and healthcare to step up, not able to say no. Conflict with self-determination. Also being proud to help. New unspoken rules to rely on. Feelings of being on shaking ground. E-learning to fresh up and increase competence, was not sufficient. Learning as they go, feel the way forward. Tacit knowledge could not be read in a book. A fear of not having the competence for the tasks. Teamwork, environment important for the nurses´ expanded role and development. |
| van Schothorst–van Roekel et al. (2021), Germany | Nurses (n = 11), hospital ward care | Qualitative case study, interviews, observations, shadowing, focus groups | The role of assistant nurses* and nurses. New nurses sought support from more experienced assistant nurses. Nurses developed a complexity measurement tool to distinguish between high/low complex patients to divide responsibility between nurses and assistant nurses. The tool did not work in practice, the assistant nurses felt undervalued when patients was considered too complex for them to take care of. Nurses was also gradually taking over/developed new coordination roles for e.g. bed occupancy at the ward from reluctant ward managers. This was complex and the nurses took support from the assistant nurses. However, the nurses later turned down the assistant nurses´ wishes of being equally responsible for coordinating care at the ward. All professionals held on to their new roles and tasks.  The nurses consider their role more complex and shaped by clinical experience. The tool disrupted organization and planning and complex patients were in novice nurses care, risking patient safety. The tool hindered teamwork. Nurses developed a hybrid role. Small changes, experiments, action and appraisals for the new roles were found positive to translate knowledge into practice and stimulate collaboration and teamwork. |
| Vitale et al. (2024), Italy | Nurses (n=403), hospital ward care | Quantitative, cohort explorative study, online questionnaire | Complex world between healthcare environment and nurses´ ability to act autonomously. The nurses´ autonomously acting depended on ward and shift.  Relationship between ward affiliation and educational levels on the ability to act autonomously, act individually/in team. |
| Willman et al. (2021), Sweden | Nurses (n=16), hospital ward care | Qualitative, focus groups | Nursing responsibilities and tasks were shifted to assistant nurses* as much as possible due to high workload. Assistant nurses did not document all things due to lack of competence. Trust in assistant nurses´ competence was essential.  Nurses missed on bedside care, and patients could be very ill, they lacked control over those who did not need complex nursing care. |
| Woldring et al. (2023), Netherlands | Nurses (n=7), hospital ward care | Qualitative, semi-structured interviews | Nurses and physicians saw a role for themselves in communication and collaboration to patients and families that was not clear nor communicated towards each other. Expectations and roles were therefore overlapping, unclear, and different. Some tasks/responsibilities were assumed to be a nursing tasks, such as providing support to families. There was unclear which information that was provided to the patient by the nurse/physician, why overlapping roles occurred.  A need for a clarification and division of the nurses´ and physicians´ roles and responsibilities in patient/family work to improve partnership. The need for building relationship and information sharing between professionals and patients/families. Respect and being aware of own and others´ roles and skills in the team. |
| Wuyts et al. (2022), Belgium | Nurses (n=259), hospital care | Quantitative, survey | Nurses need to take own decisions regarding IV-fluids in absence of a physician, at night and, urgent situations. Also, absence of IV-fluid prescription was a problem. Nurses assessed lab-results and was more aware of guidelines at the wards. More education is needed by nurses to more safely take responsibility for these tasks. Also, the teamwork/guidelines with physicians should improve teamwork to avoid errors. |

- The title assistant nurses have been used as a common term for all kinds of healthcare assistants/nurse assistants/enrolled nurses/assistant practitioner/vocational trained nurses
