# Supplementary material for: The Evolving Role of Nurses in Hospital Settings—A Scoping Review
Source: J Adv Nurs. 2025 Nov 11;82(8):7704–16. doi: 10.1111/jan.70345 (PMC13356416; doi:10.1111/jan.70345)
Supplement: Supplementary file 3 — Data S3: jan70345‐sup‐0003‐DataS3.docx. [file JAN-82-7704-s001.docx]

Attachment III: Overview of included studies’ representation in the study result

| Author/year | Navigating role ambiguity through trust and a learning community | | | Evolving the nurses´ role through adaptability, leadership and personal engagement | |
| --- | --- | --- | --- | --- | --- |
|  | Role uncertainty contributed to mistrust of competence | Managing an expanded role as a nurse | Strengthening the nurses´ role in a supportive environment | Professional growth by personal engagement and adaptive traits | Shifting towards supportive leadership and coordination |
| Agerholm et al. (2023) |  | X |  | X |  |
| Bafandeh Zendeh et al. (2022) |  | X | X | X |  |
| Bolme et al. (2021) |  | X | X | X |  |
| Carroll et al. (2024) | X |  | X | X |  |
| Chua et al. (2022) | X |  |  |  | X |
| Dúason et al. (2021) | X | X | X |  |  |
| Enggaard et al. (2024) | X |  |  |  | X |
| Enger & Andershed (2018) |  |  |  | X |  |
| Espinoza et al. (2016) |  |  | X |  | X |
| Henshall et al. (2018) | X |  |  |  | X |
| Jensen et al. (2019) |  |  |  | X | X |
| Jin et al. (2024) |  |  | X | X |  |
| Langkjaer et al. (2023) |  |  |  |  | X |
| Liang et al. (2021) |  | X |  | X | X |
| Logan et al. (2021) |  | X | X |  |  |
| Milton et al. (2022) |  |  | X |  | X |
| Mink et al. (2023) |  |  | X |  |  |
| Plantinga et al. (2024) |  |  | X |  |  |
| Sjölander et al. (2017) |  | X |  |  |  |
| Timmins et al. (2018) |  |  | X |  |  |
| Trettin et al. (2024) |  | X |  | X |  |
| van Schothorst–van Roekel et al. (2021) |  | X |  |  |  |
| Vitale et al. (2024) |  |  |  | X |  |
| Willman et al. (2021) | X |  |  |  |  |
| Woldring et al. (2023) |  |  | X |  |  |
| Wuyts et al. (2022) |  | X |  |  |  |
